# Molecular Detection of Carbapenemases in Enterobacterales: A Comparison of Real-Time Multiplex PCR and Whole-Genome Sequencing

**DOI:** 10.3390/antibiotics10060726

**Published:** 2021-06-16

**Authors:** Katja Probst, Dennis Nurjadi, Klaus Heeg, Anne-Marie Frede, Alexander H. Dalpke, Sébastien Boutin

**Affiliations:** 1 Department of Infectious Diseases, Medical Microbiology and Hygiene, University Hospital Heidelberg, 69120 Heidelberg, Germany; dennis.nurjadi@med.uni-heidelberg.de (D.N.); klaus.heeg@med.uni-heidelberg.de (K.H.); anne.frede@gmx.de (A.-M.F.); sebastien.boutin@med.uni-heidelberg.de (S.B.); 2 Institute of Medical Microbiology and Virology, University Hospital Carl Gustav Carus, 01307 Dresden, Germany; alexander.dalpke@uniklinikum-dresden.de; 3 Translational Lung Research Center Heidelberg (TLRC), German Center for Lung Research (DZL), Heidelberg University Hospital, 69120 Heidelberg, Germany

**Keywords:** antimicrobial resistance, carbapenem inactivation method, carbapenem-resistant Enterobacterales, real-time multiplex PCR, whole-genome sequencing

## Abstract

Carbapenem-resistant Enterobacterales are a growing problem in healthcare systems worldwide. While whole-genome sequencing (WGS) has become a powerful tool for analyzing transmission and possible outbreaks, it remains laborious, and the limitations in diagnostic workflows are not well studied. The aim of this study was to compare the performance of WGS and real-time multiplex PCR (RT-qPCR) for diagnosing carbapenem-resistant Enterobacterales. In this study, we analyzed 92 phenotypically carbapenem-resistant Enterobacterales, sent to the University Hospital Heidelberg in 2019, by the carbapenem inactivation method (CIM) and compared WGS and RT-qPCR as genotypic carbapenemase detection methods. In total, 80.4% of the collected isolates were identified as carbapenemase producers. For six isolates, discordant results were recorded for WGS, PCR and CIM, as the carbapenemase genes were initially not detected by WGS. A reanalysis using raw reads, rather than assembly, highlighted a coverage issue with failure to detect carbapenemases located in contigs with a coverage lower than 10×, which were then discarded. Our study shows that multiplex RT-qPCR and CIM can be a simple alternative to WGS for basic surveillance of carbapenemase-producing Enterobacterales. Using WGS in clinical workflow has some limitations, especially regarding coverage and sensitivity. We demonstrate that antimicrobial resistance gene detection should be performed on the raw reads or non-curated draft genome to increase sensitivity.

## 1. Introduction

Enterobacterales, including bacterial species such as *Citrobacter freundii*, *Escherichia coli*, *Klebsiella pneumoniae* and the *Enterobacter cloacae* complex, belong to the most common human pathogens and are able to cause a variety of infections [1,2].

In particular, infections with multidrug resistant Enterobacterales lead to high mortality since there are limited treatment options [3]. Carbapenemases are of great concern, as they are able to inactivate the last-resort drug carbapenems in addition to other beta-lactam antibiotics [3,4]. They are mostly plasmid encoded, which facilitates an easy transmission and dissemination through horizontal gene transfer [5]. Worldwide, the most common carbapenemases in Enterobacterales are KPC, NDM, VIM, IMP and OXA-48-like carbapenemases [2,6]. Another less frequent route of carbapenem resistance acquisition is via overexpression of the outer membrane efflux pumps or porin loss combined with the expression of extended-spectrum beta-lactamases or *AmpC* resistance genes [7,8].

Phenotypic screening for carbapenem resistance by Carba-NP test [9], the modified Hodge test [10] or the disc diffusion assay [11] is common in microbiology diagnostics, yet for epidemiological surveillance, high-resolution typing is useful and essential. A few real-time PCR (RT-qPCR)-based assays have been developed to detect carbapenem-resistance genes in Gram-negative bacteria [12,13,14]. However, these methods are technically limited to a certain number of targets. By contrast, whole-genome sequencing (WGS) provides more comprehensive information and thus has become a powerful tool for surveillance and outbreak investigation [15]. Although there are several studies comparing the performance of phenotypic and commercially available tests for carbapenemase detection [16,17,18], comparative studies on WGS and RT-qPCR remain scarce. Currently, the application of WGS in the clinical microbiological setting is limited to molecular typing. However, there is still an untapped potential for integrating WGS-based technologies into microbiological diagnostics. Although preparation and turnover time remains a major disadvantage for WGS, the performance and accuracy of WGS compared to those of faster nucleic acid amplification-based and simple phenotypic methods should be investigated.

Our study aimed to retrospectively evaluate the performance of WGS compared to that of RT-qPCR and phenotypic carbapenem-resistant Enterobacterales, identified by antimicrobial susceptibility testing and the carbapenem inactivation method (CIM).

## 2. Results

A total of 92 phenotypic carbapenem-resistant Enterobacterales were collected in 2019. Carbapenem-hydrolyzing activity could be detected in 74 isolates (80.4%) by CIM. These results were validated by WGS and RT-qPCR. For six isolates, different results occurred between the three methods, as carbapenemases were initially detected by CIM and PCR but not by WGS (Table 1 and Table 2). By reanalyzing the raw sequencing data and removing the coverage threshold *bla*_NDM-1_, *bla*_KPC-2_ (2x), *bla*_VIM-1_ (2x) and *bla*_OXA-48_ were identified (Table A1). For 18 isolates, all three methods revealed no carbapenemase.

**Figure 1:**
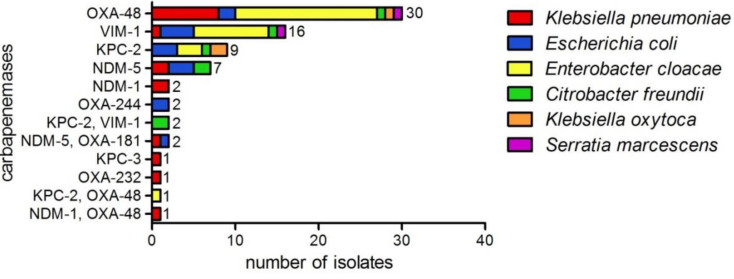
Carbapenemases detected in Enterobacterales by WGS (*n* = 74), showing phenotypic resistance to carbapenem antibiotics. *E. cloacae* (n=30), *K. pneumoniae* (*n* = 17), *E. coli* (*n* = 15), *C. freundii* (*n* = 7), *K. oxytoca* (*n* = 3) and *S. marcescens* (*n* = 2).

The predominant species of the carbapenemase producers was *E. cloacae* (*n* = 30) followed by *K. pneumoniae* (*n* = 17) and *E. coli* (*n* = 15). *C. freundii* (*n* = 7), *Klebsiella oxytoca* (*n* = 3) and *Serratia marcescens* (*n* = 2) appeared less frequently (Figure 1). OXA-48 (40.5%) was the most prevalent carbapenemase and was detected in all species in this collection. VIM-1 (21.6%) was the second most common enzyme in our study, followed by KPC-2 (12.2%) and NDM-5 (9.5%). Other carbapenemase variants, such as NDM-1, OXA-244, KPC-3 and OXA-232, were less abundant (<3.0%), and isolates harboring two carbapenemases (8.1%) occurred sporadically (Figure 1, Table A1).

## 3. Discussion

Rapid spreading of carbapenemase-producing Enterobacterales as well as outbreaks of different multidrug resistant bacteria is reported worldwide in clinical settings. For infection control and prevention of further dissemination, monitoring is necessary. Thus, we analyzed 92 phenotypically carbapenem-resistant Enterobacterales by CIM to confirm carbapenem-hydrolyzing activity. We then compared WGS and RT-qPCR to validate performance in detecting carbapenemase genes.

In total, 74 isolates were found to be carbapenemase producers (Figure 1). In six cases, discordant results occurred between WGS and the other two methods, since the carbapenemase was initially not detected by sequencing (Table 1 and Table 2 and Table A1). For analyzing WGS data, quality control is crucial, including coverage of the assembly, quality of de novo assembly and detection of potential DNA contamination. The read coverage is of particular importance, as it influences the sensitivity of sequencing [19]. In the initial assembly, we set up a limit of 25× coverage for the full genome, and contigs with a coverage <10× or smaller than 1000 bp were removed because they are potential contaminants or misassemblies. However, our study showed that true signals might be lost during the cleaning of the assembly, since the quality control parameters N50 and the coverage were in the desired range (Table A1). Low-copy number plasmids or plasmid loss during DNA extraction might have led to a low abundance of carbapenemase genes, and, thus, the antimicrobial resistance genes were not detected. While the establishment of such thresholds is crucial for genomic comparison and annotation of a draft genome, our data suggest that antimicrobial resistance gene detection should be performed on the non-curated draft genome to increase sensitivity.

Our findings on carbapenemase variants are in line with the data of the German national reference laboratory (NRL) in the years 2017–2019. In particular, *bla*_OXA-48_ was detected in all years, followed by *bla*_VIM-1_, *bla*_KPC-2_, *bla*_NDM-1_, *bla*_KPC-3_, *bla*_OXA-181_ and *bla*_NDM-5_ [20,21,22], which are detectable with our assay. However, depending on the geographic region, less frequent carbapenemase types, such as GES, GIM and IMI, can occur in Enterobacterales [20,21,22]. These genes are not included in our assay and, therefore, can lead to false-negative results. In 2019, these carbapenemases were not detected by WGS (Figure 1, Table A1). However, if the epidemiology changes, the PCR should be adapted to the new resistance situation.

The RT-qPCR provides a fast and inexpensive alternative for diagnostic labs without NGS facilities, although the PCR-based assay is limited to known targets. Compared to the RT-qPCR, WGS is an unbiased method that provides more information, such as genetic relationships and the full resistome. Besides the presence or absence of known resistance genes, novel resistance genes can be identified in phenotypic resistant isolates by WGS [23]. However, the analysis is more complex, and, therefore, bioinformatics expertise is needed.

## 4. Materials and Methods

### 4.1. Bacterial Isolates

Clinical samples and rectal swabs were screened for carbapenem-resistant Enterobacterales at the Department of Infectious Diseases, Medical Microbiology, University Hospital Heidelberg in 2019. During routine diagnostics, 92 Enterobacterales showing phenotypic resistance to meropenem and imipenem were collected. Non-duplicate strains were obtained from 79 patients. Multiple isolates (*n* = 13) from the same patient were included in the study due to different bacterial species as determined by MALDI TOF MS (Bruker Daltonics GmbH & Co. KG, Bremen, Germany). The antibiotic susceptibility was tested by the VITEK-2 system (bioMérieux Deutschland GmbH, Nürtingen, Germany) and evaluated according to the valid EUCAST guidelines in the respective year (v 9.0). The isolates were stored at −20 °C until usage.

### 4.2. Carbapenem Inactivation Method

CIM was performed, as described elsewhere [24], to examine whether the carbapenem-resistant isolates, identified by antimicrobial susceptibility testing, are able to hydrolyze carbapenem antibiotics.

### 4.3. DNA Extraction

The isolates were regrown on BD™ Columbia Agar with 5% Sheep Blood (Becton Dickinson GmbH, Heidelberg, Germany) at 37 °C. DNA for WGS and RT-qPCR was extracted using the DNeasy Blood and Tissue Kit (Qiagen GmbH, Hilden, Germany) according to the manufacturer’s protocol.

### 4.4. Multiplex Real-Time PCR

The assay based on hydrolysis probes consists of two multiplex PCRs for the detection of *bla*_NDM_, *bla*_KPC_, *bla*_VIM_ and *bla*_IMP_, and *bla*_OXA-23_-like, *bla*_OXA-40/24_-like, *bla*_OXA-58_-like and *bla*_OXA-48_-like, respectively. Amplification and detection were performed on the BD MAX™ system, using the protocol for the PCR-only mode, as described elsewhere [25].

### 4.5. Whole-Genome Sequencing

WGS was performed on the MIseq instrument (2 × 300 bp), using the Nextera DNA Flex Library Prep Kit (Illumina) for preparing sequencing libraries. Quality control of the raw sequences, assembly and curation (contigs >1000bp and >10× coverage) were performed as described elsewhere [26]. The databases ResFinder 3.0, ARG-ANNOT and CARD-NCBI-BARRGD using ABRIcate (https://git.lumc.nl/bvhhornung/antibiotic-resistancepipeline/tree/master/tools/abricate, accessed on 10 June 2020) were used to determine the resistance genes as previously described [27].

## 5. Conclusions

Whole-genome sequencing is a powerful tool with high molecular resolution, giving information about bacterial species, plasmid replicon types and the whole resistance pattern, which is needed for surveillance of transmission and outbreak investigation. Real-time PCR is faster but provides less information and cannot detect new carbapenemases that are not included in the panel, which is a general drawback of PCR-based assays. Nevertheless, the additional use of PCR and/or CIM for carbapenemase detection in Enterobacterales was beneficial in our study to ensure high sensitivity, as some carbapenemases would have remained undetected by WGS due to coverage issues.

## 6. Patents

K.P., K.H. and A.H.D. have a patent (No. 20203612.5) pending.

## Figures and Tables

**Table 1:** Comparison of phenotypic and genotypic carbapenemase detection in Enterobacterales by CIM, RT-qPCR and WGS.

		CIM	
		Positive	Negative
RT-qPCR	positive negative	74 0	0 18
WGS	positive negative	70(74) ^1^ 4(0) ^1^	0 18

^1^ After reanalyzing the raw sequencing data.

**Table 2:** Comparison of genotypic carbapenemase detection in Enterobacterales by WGS and RT-qPCR.

		WGS	
		Positive	Negative
RT-qPCR	positive negative	68(74) ^1^ 0	6(0) ^1^ 18

^1^ After reanalyzing the raw sequencing data.

## Data Availability

Bio project PRJNA634442.

## Appendix A

**Table A1:** Phenotypic carbapenem-resistant Enterobacterales collected in 2019, analyzed by CIM, RT-qPCR and WGS. Quality control parameters for WGS: coverage and N50.

Sample ID	Species	CIM	RT-qPCR	WGS	WGS Reanalyzed	Coverage	N50
KE9539	*E. coli*	positive	*bla*_KPC_	*bla*_KPC-2_		48	535,993
KE9246	*E. coli*	positive	*bla*_KPC_	*bla*_KPC-2_		53	135,761
KE9526	*E. cloacae*	positive	*bla*_KPC_	*bla*_KPC-2_		52	363,822
KE9478	*E. cloacae*	positive	*bla*_KPC_	*bla*_KPC-2_		96	363,822
BK31926	*E. coli*	positive	*bla*_KPC_	*bla*_KPC-2_		29	120,862
KE9621	*K. pneumoniae*	positive	*bla*_KPC_	*bla*_KPC-3_		35	386,401
KE9498	*C. freundii*	positive	*bla*_KPC_	*bla*_KPC-2_		31	200,582
KE9038	*K. oxytoca*	positive	*bla*_KPC_	*bla*_KPC-2_		50	285,607
KE9326	*K. oxytoca*	positive	*bla*_KPC_	negative	*bla*_KPC-2_	42	109,274
KE9511	*C. freundii*	positive	*bla*_KPC_, *bla*_VIM_	*bla*_KPC-2_, *bla*_VIM-1_		30	198,406
KE9378	*C. freundii*	positive	*bla*_KPC_, *bla*_VIM_	*bla*_KPC-2_	*bla*_KPC-2_, *bla*_VIM-1_	39	201,178
KE9132	*E. cloacae*	positive	*bla*_KPC_	*bla*_KPC-2_		49	363,822
KE9520	*K. pneumoniae*	positive	*bla*_NDM_	*bla*_NDM-5_		53	186,575
KE9434	*K. pneumoniae*	positive	*bla*_NDM_	*bla*_NDM-5_		34	292,061
KE9521	*E. coli*	positive	*bla*_NDM_, *bla*_OXA-48_-like	*bla*_NDM-5_, *bla*_OXA-181_		61	106,471
KE9395	*E. coli*	positive	*bla*_NDM_	*bla*_NDM-5_		54	94,083
KE9433	*E. coli*	positive	*bla*_NDM_	*bla*_NDM-5_		36	214,212
KE9636	*K. pneumoniae*	positive	*bla*_NDM_, *bla*_OXA-48_-like	*bla*_NDM-1_, *bla*_OXA-48_		27	383,090
KE9616	*C. freundii*	positive	*bla*_NDM_	*bla*_NDM-5_		50	186,958
KE9522	*E. coli*	positive	*bla*_NDM_	*bla*_NDM-5_		103	269,697
KE9593	*K. pneumoniae*	positive	*bla*_NDM_, *bla*_OXA-48_-like	*bla*_NDM-5_, *bla*_OXA-181_		38	296,725
D3014	*C. freundii*	positive	*bla*_NDM_	*bla*_NDM-5_		36	186,959
KE9449	*K. pneumoniae*	positive	*bla*_NDM_	negative	*bla*_NDM-1_	25	220,843
KE9500	*K. pneumoniae*	positive	*bla*_NDM_	*bla*_NDM-1_		33	536,321
KE9382	*E. cloacae*	positive	*bla*_OXA-48_-like	*bla*_OXA-48_		27	374,725
KE9492	*K. pneumoniae*	positive	*bla*_OXA-48_-like	*bla*_OXA-232_		30	242,997
KE9629	*E. coli*	positive	*bla*_OXA-48_-like	*bla*_OXA-244_		33	238,467
KE9025	*E. cloacae*	positive	*bla*_OXA-48_-like	*bla*_OXA-48_		49	272,750
KE9469	*E. cloacae*	positive	*bla*_OXA-48_-like	*bla*_OXA-48_		76	374,315
KE9472	*E. cloacae*	positive	*bla*_OXA-48_-like	*bla*_OXA-48_		98	382,653
KE9424	*K. pneumoniae*	positive	*bla*_OXA-48_-like	*bla*_OXA-48_		36	184,292
KE9499	*E. cloacae*	positive	*bla*_OXA-48_-like	*bla*_OXA-48_		66	486,681
KE9400	*K. pneumoniae*	positive	*bla*_OXA-48_-like	*bla*_OXA-48_		45	208,351
KE9468	*E. cloacae*	positive	*bla*_OXA-48_-like	*bla*_OXA-48_		80	383,026
KE9638	*E. coli*	positive	*bla*_OXA-48_-like	*bla*_OXA-244_		37	156,925
KE9493	*E. cloacae*	positive	*bla*_OXA-48_-like, *bla*_KPC_	*bla*_OXA-48_	*bla*_KPC-2_, *bla*_OXA-48_	44	530,933
KE9456	*K. oxytoca*	positive	*bla*_OXA-48_-like	*bla*_OXA-48_		28	223,596
KE9443	*K. pneumoniae*	positive	*bla*_OXA-48_-like	*bla*_OXA-48_		27	225,118
KE9354	*E. cloacae*	positive	*bla*_OXA-48_-like	*bla*_OXA-48_		66	486,663
BK32270	*E. coli*	positive	*bla*_OXA-48_-like	*bla*_OXA-48_		35	117,967
KE9626	*E. coli*	positive	*bla*_OXA-48_-like	*bla*_OXA-48_		53	196,578
KE9208	*S. marcescens*	positive	*bla*_OXA-48_-like	*bla*_OXA-48_		58	2,797,497
D2902	*E. cloacae*	positive	*bla*_OXA-48_-like	*bla*_OXA-48_		64	302,960
KE9541	*K. pneumoniae*	positive	*bla*_OXA-48_-like	*bla*_OXA-48_		47	427,613
KE9554	*C. freundii*	positive	*bla*_OXA-48_-like	*bla*_OXA-48_		39	165,554
KE9338	*E. cloacae*	positive	*bla*_OXA-48_-like	*bla*_OXA-48_		47	374,725
KE9355	*E. cloacae*	positive	*bla*_OXA-48_-like	*bla*_OXA-48_		109	486,681
KE9328	*K. pneumoniae*	positive	*bla*_OXA-48_-like	*bla*_OXA-48_		43	274,145
KE9510	*E. cloacae*	positive	*bla*_OXA-48_-like	*bla*_OXA-48_		31	491,022
D3070	*E. cloacae*	positive	*bla*_OXA-48_-like	*bla*_OXA-48_		44	372,768
KE9428	*E. cloacae*	positive	*bla*_OXA-48_-like	*bla*_OXA-48_		36	486,663
KE9527	*E. cloacae*	positive	*bla*_OXA-48_-like	negative	*bla*_OXA-48_	27	339,153
D3018	*K. pneumoniae*	positive	*bla*_OXA-48_-like	*bla*_OXA-48_		25	473,650
D3082	*E. cloacae*	positive	*bla*_OXA-48_-like	*bla*_OXA-48_		62	486,663
KE9637	*K. pneumoniae*	positive	*bla*_OXA-48_-like	*bla*_OXA-48_		36	876,600
D3081	*E. cloacae*	positive	*bla*_OXA-48_-like	*bla*_OXA-48_		85	383,026
EX1012	*K. pneumoniae*	positive	*bla*_OXA-48_-like	*bla*_OXA-48_		39	223,327
D3078	*E. cloacae*	positive	*bla*_OXA-48_-like	*bla*_OXA-48_		54	486,828
KE9366	*E. coli*	positive	*bla*_VIM_	*bla*_VIM-1_		38	215,473
KE9563	*E. cloacae*	positive	*bla*_VIM_	*bla*_VIM-1_		35	377,920
KE9409	*E. cloacae*	positive	*bla*_VIM_	*bla*_VIM-1_		46	486,118
KE9414	*E. cloacae*	positive	*bla*_VIM_	*bla*_VIM-1_		38	161,463
KE9365	*K. pneumoniae*	positive	*bla*_VIM_	*bla*_VIM-1_		32	232,474
KE9538	*S. marcescens*	positive	*bla*_VIM_	*bla*_VIM-1_		40	1,130,420
KE9585	*E. cloacae*	positive	*bla*_VIM_	*bla*_VIM-1_		25	287,090
KE9559	*C. freundii*	positive	*bla*_VIM_	*bla*_VIM-1_		41	163,976
KE9549	*E. coli*	positive	*bla*_VIM_	*bla*_VIM-1_		47	279,067
KE9548	*E. cloacae*	positive	*bla*_VIM_	*bla*_VIM-1_		46	230,814
KE9579	*E. coli*	positive	*bla*_VIM_	*bla*_VIM-1_		39	112,495
KE9474	*E. cloacae*	positive	*bla*_VIM_	*bla*_VIM-1_		38	290,132
KE9462	*E. cloacae*	positive	*bla*_VIM_	*bla*_VIM-1_		33	502,528
KE9560	*E. cloacae*	positive	*bla*_VIM_	*bla*_VIM-1_		40	290,117
KE9575	*E. cloacae*	positive	*bla*_VIM_	*bla*_VIM-1_		38	389,538
KE9536	*E. coli*	positive	*bla*_VIM_	negative	*bla*_VIM-1_	44	377,920
D2923	*E. cloacae*	negative	negative	negative		58	203,439
KE9347	*E. cloacae*	negative	negative	negative		79	439,426
KE9591	*E. cloacae*	negative	negative	negative		47	279,225
KE9576	*E. coli*	negative	negative	negative		27	228,481
KE9599	*E. coli*	negative	negative	negative		37	281,932
KE9623	*E. coli*	negative	negative	negative		50	93,960
KE9633	*K. aerogenes*	negative	negative	negative		40	495,847
KE9068	*C. freundii*	negative	negative	negative		46	176,242
KE8986	*E. cloacae*	negative	negative	negative		47	230,847
KE9344	*E. cloacae*	negative	negative	negative		48	235,301
KE9475	*E. cloacae*	negative	negative	negative		40	208,042
KE9083	*E. coli*	negative	negative	negative		57	208,544
KE9425	*K. aerogenes*	negative	negative	negative		48	902,223
KE9614	*K. aerogenes*	negative	negative	negative		62	429,809
D3017	*K. pneumoniae*	negative	negative	negative		27	232,937
KE9095	*K. pneumoniae*	negative	negative	negative		66	237,389
KE9171	*K. pneumoniae*	negative	negative	negative		54	481,561
KE9039	*S. marcescens*	negative	negative	negative		40	1,228,444
